# Longitudinal assessment of cardio-respiratory fitness among Indian patients with type 2 diabetes mellitus

**DOI:** 10.6026/9732063002001261

**Published:** 2024-10-31

**Authors:** Sushmita Palia, Mounish Reddy, Shreya Seira Honarius, Madhurika Jalakam, Ruchi Kothari, Mayur Wanjari, Labdhi Sangoi, Ravi Sangoi

**Affiliations:** 1 Department of Cardiology, All India Institute of Medical Science, Guntur District, Mangala Giri, Andhra Pradesh 522503, India; 2 Department of Emergency Medicine, Leeds Teaching Hospitals NHS Trust, Leeds, United Kingdom; 3 Resident Doctor (Cardiology) St. Johns Medical College, Bangalore, India; 4 SHO, ITU Department, Queen Elizabeth The Queen Mother Hospital, Kent CT9 4AN East Kent Hospitals University NHS Foundation Trust, St Peters Road, Margate, England; 5 Department of Physiology, Mahatma Gandhi Institute of Medical Sciences, Sevagram, Wardha, Maharashtra, India; 6 Department of Research, Datta Meghe Institute of Higher Education & Research (DMIHER), Sawangi, Maharashtra, India; 7 Department of Research, Government Medical College, Jalna, Maharashtra; 8 Department of Internal Medicine, Government Medical College, Baramati, India

**Keywords:** Diabetes mellitus, cardiorespiratory, longitudinal study, autonomic dysfunction, sports physiology

## Abstract

Type 2 Diabetes Mellitus (T2DM) is associated with many complications, including cardiovascular and autonomic dysfunctions. Cardiorespiratory fitness as estimated by maximal oxygen uptake (VO2 max) is a very powerful predictor of cardiovascular health. Therefore, it is of interest to measure the cardiorespiratory parameters in T2DM patients for diagnosing autonomic dysfunction and to follow the changes over time. Baseline and follow-up cardiorespiratory fitness parameters among patients of Central India suffering from T2DM and its effectiveness to lifestyle modifications for these parameters are done. This hospital-based longitudinal study was conducted on 600 patients between the age group of 30 and 65 years diagnosed with T2DM. Patients were recruited from the Sports Physiology Laboratory, Department of Physiology, Mahatma Gandhi Institute of Medical Sciences, Sevagram, Maharashtra. Baseline measurements of VO2 max, HRV, and other cardiorespiratory variables were taken with a motorized treadmill using Lab Chart. Then, lifestyle counselling was undertaken for the participants and the same parameters were reassessed one year later. Statistical package SPSS version 23 was used during data analysis. After one year of interventions, the improvements at the end of one year include those of VO2 max and HRV. The mean VO2 max improved from 25.4 ± 5.2 to 30.1 ± 4.8 ml/kg/min while the probability was less than 0.001. The main indices of HRV showed improved autonomic balance along with enhanced parasympathetic activity. Combining lifestyle interventions with regular monitoring of cardiorespiratory fitness and HRV can, indeed significantly improve cardiovascular health in T2DM patients. This study calls for the inclusion of fitness assessments in everyday clinical care for diabetes.

## Background:

Diabetes mellitus is considered a public health issue in the country as it has been surging over time, and above 62 million have diagnosed with the disease in India [1]. According to the data, the number of cases of diabetes is estimated to increase up to 79.4 million in 2030, where more than 90% are going to type 2 Diabetes Mellitus that is responsible for more than 90% of types in this population [2]. T2DM is not only a metabolic disease but also significantly promotes cardiovascular complications due to autonomic dysfunction [3]. In this context, the autonomic nervous system, which controls cardiovascular function, has been affected with dysfunction often being one of the earliest manifestations of augmented cardiovascular risk in patients with diabetes [4]. Heart Rate Variability (HRV) is a non-invasive marker of the balance between sympathetic and parasympathetic branches of ANS. Lower HRV is linked with increased risk of cardiovascular events and mortality among T2DM patients [5]. A second critical measure for cardiovascular health assessment is maximal oxygen uptake, that is commonly stated as VO2 max, an index for cardiorespiratory fitness and a highly sensitive predictor of cardiovascular outcomes. Lower values of VO2max have been reported to be associated with higher cardiovascular morbidity and mortality [6]. Most of the previous studies on cardiorespiratory fitness and HRV in diabetes have focused on particular populations, thus excluding the diversified population of the Indian subcontinent [7]. Furthermore, limited literature is available that addresses both VO2 max and HRV in T2DM patients [8]. Therefore, it is of interest for bridging the gap by longitudinally assessing the cardiorespiratory fitness and HRV of T2DM patients in Central India and determining the effect of lifestyle modifications on these parameters over a three-year period [9]. Therefore, it is of interest to report the longitudinal assessment of cardio respiratory fitness among Indian patients with type 2 diabetes mellitus.

## Methodology:

## Study design:

A hospital-based, long-term, observational study conducted over a period of three years from 1st October 2015 to 1st October 2018. The research was undertaken in the Sports Physiology Laboratory, Department of Physiology, Mahatma Gandhi Institute of Medical Sciences, Sevagram, Wardha, Maharashtra. Table 1 shows Demographic characteristics of study participants.

## Study Population:

Altogether, 600 patients aged between 30 and 65 years, who were diagnosed as having T2DM, were recruited for this study. It is the criterion of selection wherein all the diagnosed cases were confirmed by a consultant physician, were willing to give informed consent, and who were ready to come under the study protocol.

## Inclusion criteria:

[1] Patient of the age group 30-65 years with a diagnosis of T2DM

[2] Willingness to participate and to give informed consent

[3] No contra-indications for the performance of treadmill exercise testing

## Exclusion criteria:

[1] Patients with a history of cardiovascular, respiratory, or psychiatric disorders

[2] Patients having any comorbidity that can interfere with exercise testing

[3] Patients on medicines that may influence HRV, *e.g.*, beta-blockers

## Baseline assessment:

This involved the collection of baseline data at the beginning of the study, which consisted of demographic details, clinical history, and anthropometric measurements. These included height, weight, and waist circumference. The cardiovascular and respiratory fitness parameters assessed included VO2 max, respiratory rate, tidal volume, and heart rate variability. The ECG signal was measured using the Power Lab System in conjunction with the Lab Chart software. VO2 max was obtained through a graded exercise test on the motorized treadmill until participants experienced volitional fatigue. Heart rate variability was recorded for 5 minutes in a supine position using a wireless ECG sensor.

## Lifestyle modification counselling:

Lifestyle modification counselling was provided to all participants, including dietary advice and regular physical activity and techniques for stress management. The counselling was refreshed at pre-scheduled intervals during the follow-up period.

## Follow-Up assessment:

At the end of one year, the parameters had once again been evaluated by comparable testing. Participants had a repeat of graded exercise test and HRV analysis. Their changes in cardiorespiratory fitness and HRV were measured to evaluate the changes in lifestyle modification.

## Data analysis:

Data were entered into a secure database and analysed using SPSS version 23. Continuous variables were expressed as mean ± standard deviation. The paired t-tests were used to compare values before and after the intervention. The Pearson's correlation coefficient was utilized to examine the association between VO2 max and HRV. A p-value of <0.05 was considered statistically significant.

## Ethical consideration:

This study conforms to the Declaration of Helsinki. Ethical permission has been taken from the Institutional Ethics Committee of the Mahatma Gandhi Institute of Medical Sciences. (Ref. no: MGIMS/IEC/PHY/09/2019) Informed consent was taken from all volunteers.

## Results:

Table 2 shows Baseline cardiopulmonary parameters of participants. Table 3 shows follow up details of the participants. Table 4 shows comparison of baseline and follow-up parameters. Table 5 shows changes in cardiorespiratory parameters. Table 6 shows changes in Sympathovagal i.e HRV parameters. Table 7 shows correlation between the study parameters. Table 8 shows lifestyle modifications. Table 9 shows changes in cardiovascular risk factors.

The average age of participants was 52.3 years, with a balanced gender distribution. The mean BMI indicates that most participants were overweight, which is common in T2DM patients. Baseline measurements show that participants had a low VO2 max, indicating reduced cardiorespiratory fitness. Elevated resting heart rate and blood pressure values are consistent with autonomic dysfunction in T2DM. Significant improvements were observed in all measured parameters following lifestyle modifications, with notable increases in VO2 max and reductions in resting heart rate and blood pressure. The results indicate significant improvements in all measured parameters following lifestyle interventions. The increase in VO2 max and reductions in resting heart rate and blood pressure suggest improved cardiorespiratory fitness and better autonomic balance in the study participants. All age groups showed significant improvements in VO2 max following lifestyle modifications, with younger participants demonstrating a relatively greater increase in aerobic capacity. Significant improvements were observed in all HRV parameters, indicating enhanced autonomic function with increased parasympathetic activity and reduced sympathetic dominance following the intervention. Participants in all BMI categories showed significant improvements in VO2 max and HRV parameters, with greater changes observed in those with normal BMI compared to overweight and obese categories. There were significant positive correlations between VO2 max and HRV parameters, indicating that improvements in aerobic capacity are associated with better autonomic function. The negative correlation with the LF/HF ratio suggests reduced sympathetic dominance with increased aerobic capacity. Most of the participants adhered to dietary recommendations and regular physical activity, while adherence to stress management practices was lower. Complete adherence was achieved by 50% of the participants. Significant improvements were observed in all measured cardiovascular risk factors, indicating that lifestyle modifications not only improved cardiorespiratory fitness but also contributed to better glycaemic control and lipid profile.

## Discussion:

The results in this longitudinal study reveal critical impacts of lifestyle interventions upon cardiorespiratory fitness and autonomic function in T2DM patients [10]. VO2 max and HRV measures improved with age where youths demonstrated greater relative increases in aerobic capacity [11]. This reflects that early intervention by lifestyle modifications would present even greater benefits in patients with T2DM [12]. In support of high cardiorespiratory fitness, all the HRV parameters showed a positive correlation with VO2 max [13]. Lifestyle intervention-compliant individuals demonstrated significant reductions in cardiovascular risk factors, which included fasting blood glucose, HbA1c, and the lipid profile parameters [14]. This was consistent with other studies, showing that lifestyle modification results in the reduction of incidence of cardiovascular complications among diabetic patients [15]. However, for almost half of the participants, full adherence to the lifestyle modification occurred, which seems to indicate that better support systems and follow-up would be required for enhanced levels of adherence to take place [16]. This suggests that possibly less costly and more attractive stress management techniques may need to be introduced to enhance participation since the uptake of practices such as yoga remains low [17]. The improvements observed in overweight and obese subjects, while statistically highly significant, were quantitatively smaller than in the group with normal BMI [18]. This factor alone draws attention to the difficulties that need to be overcome to optimize cardiorespiratory fitness in people with a higher BMI [19]. Tailor-made interventions based on weight reduction may be necessary to gain better results in these populations. Such studies do have some limitations. Self-reported adherence to lifestyle changes is likely to be prone to biases. The single-centre trial will limit the generalizability of results to a greater degree. Larger, multi-centre, longer follow-up trials with objective measures of adherence could validate such findings in future studies. As also shown by previous studies compliance with moderate type of physical activity can significantly improve the fasting and postprandial blood sugar, apart from reduction in weight of T2DM patients which can be delved more into in future studies [20, 21-22].

## Conclusion:

Data shows that lifestyle modifications, including diet, regular physical activity, and stress management, significantly improve cardiorespiratory fitness, autonomic function and cardiovascular risk factors in T2DM patients. Regular monitoring and tailored interventions should be integrated into the routine clinical management of diabetes to prevent complications and improve quality of life. Further research is needed to explore the long-term effects of these interventions and to develop strategies to enhance adherence and engagement among diabetic patients.

## Figures and Tables

**Table 1:** Demographic characteristics of study participants

**Variable**	**Mean ± SD**
Age (years)	52.3 ± 9.8
Gender (M/F)	320/280
BMI (kg/m^2^)	26.5 ± 3.4
Waist Circumference (cm)	95.2 ± 8.7

**Table 2:** Baseline cardiopulmonary parameters

**Parameter**	**Mean ± SD**
Resting Heart Rate (bpm)	82.4 ± 10.5
Systolic Blood Pressure (mmHg)	134.5 ± 15.6
Diastolic Blood Pressure (mmHg)	85.2 ± 9.4
Respiratory Rate (breaths/min)	18.6 ± 2.3
VO2 Max (ml/kg/min)	25.4 ± 5.2

**Table 3:** Follow-Up Cardiopulmonary Parameters (1 Year)

**Parameter**	**Mean ± SD**
Resting Heart Rate (bpm)	78.2 ± 9.8
Systolic Blood Pressure (mmHg)	128.6 ± 13.4
Diastolic Blood Pressure (mmHg)	81.5 ± 8.6
Respiratory Rate (breaths/min)	17.4 ± 2.0
VO2 Max (ml/kg/min)	30.1 ± 4.8

**Table 4:** Comparison of Baseline and Follow-Up Parameters

**Parameter**	**Baseline Mean ± SD**	**Follow-Up Mean ± SD**	**p-value**
Resting Heart Rate (bpm)	82.4 ± 10.5	78.2 ± 9.8	<0.001*
Systolic Blood Pressure (mmHg)	134.5 ± 15.6	128.6 ± 13.4	<0.001*
Diastolic Blood Pressure (mmHg)	85.2 ± 9.4	81.5 ± 8.6	<0.001*
Respiratory Rate (breaths/min)	18.6 ± 2.3	17.4 ± 2.0	<0.001*
VO2 Max (ml/kg/min)	25.4 ± 5.2	30.1 ± 4.8	<0.001*

**Table 5:** Changes in VO2 Max by Age Group

**Age Group (years)**	**Baseline VO_2_ Max (ml/kg/min) Mean ± SD**	**Follow-Up VO_2_ Max (ml/kg/min) Mean ± SD**	**p-value**
30-40	27.1 ± 4.6	32.8 ± 4.2	<0.001*
41-50	26.3 ± 4.9	31.5 ± 4.5	<0.001*
51-60	24.0 ± 5.1	28.7 ± 4.8	<0.001*
>60	22.5 ± 5.4	26.9 ± 4.7	<0.001*

**Table 6:** Changes in Heart Rate variability (HRV) parameters

**HRV Parameter**	**Baseline Mean ± SD**	**Follow-Up Mean ± SD**	**p-value**
SDNN (ms)	32.5 ± 11.2	42.8 ± 13.7	<0.001*
RMSSD (ms)	20.4 ± 7.5	28.1 ± 9.8	<0.001*
LF (ms^2^)	420 ± 180	530 ± 210	0.02*
HF (ms^2^)	380 ± 170	480 ± 190	0.01*
LF/HF Ratio	1.1 ± 0.5	1.0 ± 0.4	0.05*

**Table 7:** Correlation between VO2 Max and HRV parameters

**Parameter**	**VO_2_ Max Correlation Coefficient ®**	**p-value**
SDNN	0.58	<0.001*
RMSSD	0.55	<0.001*
LF	0.49	0.001*
HF	0.52	<0.001*
LF/HF Ratio	-0.45	0.001*

**Table 8:** Adherence to lifestyle modifications

**Parameter**	**Number of Participants (%)**
Adherence to Diet Plan	520 (86.7%)
Regular Physical Activity (≥ 3 times/week)	480 (80%)
Stress Management Practice (*e.g.*, Yoga)	350 (58.3%)
Complete Adherence (All Parameters)	300 (50%)

**Table 9:** Changes in cardiovascular risk factors

**Risk Factor**	**Baseline Mean ± SD**	**Follow-Up Mean ± SD**	**p-value**
Fasting Blood Glucose (mg/dL)	150.5 ± 25.3	130.2 ± 20.4	<0.001*
HbA1c (%)	7.8 ± 1.1	6.9 ± 0.9	<0.001*
Total Cholesterol (mg/dL)	220.4 ± 35.2	200.1 ± 30.3	<0.001*
LDL Cholesterol (mg/dL)	140.6 ± 28.4	125.5 ± 25.2	<0.001*
HDL Cholesterol (mg/dL)	45.2 ± 8.7	48.6 ± 9.1	<0.001*
Triglycerides (mg/dL)	180.4 ± 40.8	160.2 ± 35.5	<0.001*
